# Effects of Anodal Transcranial Direct Current Stimulation on Visually Guided Learning of Grip Force Control

**DOI:** 10.3390/biology4010173

**Published:** 2015-03-02

**Authors:** Tamas Minarik, Paul Sauseng, Lewis Dunne, Barbara Berger, Annette Sterr

**Affiliations:** 1 Department of Psychology, Ludwig-Maximilian University, Munich, Leopoldstr. 13, Munich 80802, Germany; E-Mails: tamas.minarik@psy.lmu.de (T.M.); paul.sauseng@psy.lmu.de (P.S.); barbara.berger@psy.lmu.de (B.B.); 2 School of Psychology, University of Surrey, Guildford GU2 7XH, UK; E-Mail: ld00139@surrey.ac.uk; 3 Department of Neurology, University of Sao Paulo, São Paulo, SP 01246903, Brazil

**Keywords:** power grip, force, neurofeedback, motor cortex

## Abstract

Anodal transcranial Direct Current Stimulation (tDCS) has been shown to be an effective non-invasive brain stimulation method for improving cognitive and motor functioning in patients with neurological deficits. tDCS over motor cortex (M1), for instance, facilitates motor learning in stroke patients. However, the literature on anodal tDCS effects on motor learning in healthy participants is inconclusive, and the effects of tDCS on visuo-motor integration are not well understood. In the present study we examined whether tDCS over the contralateral motor cortex enhances learning of grip-force output in a visually guided feedback task in young and neurologically healthy volunteers. Twenty minutes of 1 mA anodal tDCS were applied over the primary motor cortex (M1) contralateral to the dominant (right) hand, during the first half of a 40 min power-grip task. This task required the control of a visual signal by modulating the strength of the power-grip for six seconds per trial. Each participant completed a two-session sham-controlled crossover protocol. The stimulation conditions were counterbalanced across participants and the sessions were one week apart. Performance measures comprised time-on-target and target-deviation, and were calculated for the periods of stimulation (or sham) and during the afterphase respectively. Statistical analyses revealed significant performance improvements over the stimulation and the afterphase, but this learning effect was not modulated by tDCS condition. This suggests that the form of visuomotor learning taking place in the present task was not sensitive to neurostimulation. These null effects, together with similar reports for other types of motor tasks, lead to the proposition that tDCS facilitation of motor learning might be restricted to cases or situations where the motor system is challenged, such as motor deficits, advanced age, or very high task demand.

## 1. Introduction

Visually-guided movements are essential for everyday functioning. Such movements are based on visuomotor integration and range from simple reaching towards an object to complex abilities like driving. Visuomotor integration requires the complex interaction between the visual and the motor systems in order to use visual information to guide motor output and also to use movement information to influence the processing of visual information. Given the constant necessity of such actions and skills, the ability to maintain them and to learn new ones throughout life seems obvious. However, stroke, Parkinson’s disease, traumatic brain injury and many other disorders can cause temporary or lasting motor function deficits, in which such visuomotor function and learning often become impaired [1]. Recent studies suggest that the recovery from these impairments might be aided by non-invasive neurostimulation methods [2,3]. In particular, transcranial Direct Current Stimulation (tDCS) has been shown to facilitate motor learning, and to have positive therapeutic effects in patients with motor deficits [4,5]. The application of weak electric stimulation over selected brain areas alters cortical excitability [6,7]. tDCS is thereby thought to aid neuroplasticity and functional/structural reorganization of the brain by modifying synaptic connections via the induction of long-term potentiation (LTP) and long-term depression (LTD), as well as the modulation of the neuronal resting membrane potential [8,9].

Recovery from stroke can benefit from anodal tDCS applied over the primary motor cortex (M1) of the affected hemisphere, but also from cathodal tDCS over the contralesional M1 [10,11,12]. Therapeutic tDCS application has further been shown to improve motor function in patients with morbus Parkinson [13]. These outcomes are very encouraging; however, overall the evidence-base for the therapeutic tDCS benefits is mixed with often small and variable treatment effects [14,15,16]. Moreover, tDCS effects on motor learning and motor control in healthy populations also appear to be inconclusive [17]. For instance, Antal *et al.* [18] found that 10 min of 1mA anodal tDCS applied over the contralateral M1 (and also over the primary visual cortex) during a visuomotor tracking task enhanced performance in healthy volunteers. The enhancement was thereby most pronounced in the early phase of learning, suggesting that tDCS facilitates the early learning processes in particular (see also Vollmann *et al.* [19] for similar results). However, a similar tDCS stimulation protocol, applied in a comparable visuomotor tracking task, failed to induce facilitation effects in the study by Saiote *et al.* [20]. Using a simple visuomotor task, Galea *et al.* [21] further found that 15 min of 2 mA anodal tDCS over the contralateral M1 improved skill retention between the sessions; early learning was thereby only facilitated when tDCS was applied over the cerebellum but not when tDCS was applied to the contralateral M1. Retention, but not early learning, also improved in a pinch-force task exerted with the non-dominant hand in response to 20 min of 1 mA anodal tDCS applied over the contralateral M1 in a sample of healthy young adults [22].

Previous studies by our group have employed a visually-guided motor tracking task involving the power grip. Designed to be suitable for the study of motor plasticity in healthy persons and patients with upper-limb dysfunction, this task has been shown to induce changes in the neural activation observed through event-related potentials [23] as well as learning indexed by changes in the mu rhythm and connectivity [24]. Functional magnetic resonance imaging (fMRI) studies further showed clear and robust activations in the visuomotor network which were modulated by task demand [25]. The task requires fine and near-isometric adjustments of power-grip output to track a visually presented and continuously changing signal on screen for several seconds. Changes in task performance over time are presumably mediated, at least in part, by the motor system and should henceforth be susceptible to anodal tDCS stimulation over M1. The present study therefore examined whether learning and performance in this task can be enhanced by anodal tDCS applied over the contralateral M1. Based on the consolidated review of the literature, a protocol with 20 min of 1 mA stimulation (active tDCS) and 20 min of tDCS sham stimulation, applied in a counterbalanced within-subjects design, was used. Participants completed the task during the 20 min stimulation phase and a 20 min afterphase (*i.e.*, 40 min in total). We hypothesized that task performance would increase over the 40 min of task execution for both stimulation conditions (active tDCS and sham), with tDCS effects being evidenced by significantly greater performance gains in the stimulation condition compared to sham stimulation. Retention effects would be indicated by sustained performance between sessions for active tDCS stimulation but not for sham stimulation. We further assumed that tDCS effects would be more pronounced during runs requiring more refined control of the power grip (hard condition) compared to an easy condition requiring less frequent and slower grip force modulations.

## 2. Experimental Section

### 2.1. Participants

Twenty-six right-handed students (22 female, 4 male) were recruited from the University of Surrey. Due to technical issues, data obtained from four participants were incomplete and hence excluded from further analyses. The final sample comprised 22 participants (20 female; *M*_age_ = 19.85, *SD*_age_ = 2.13, range 18–23). All participants were right-hand dominant. Twenty-one participants had scores on the Edinburgh Handedness Inventory of ≥60 (EHI); one participant scored 58. Participants reported no neurological condition or contraindication to tDCS and all had normal or corrected vision. The study received ethical approval from the University of Surrey Ethics Committee. Written informed consent was obtained prior to participation.

### 2.2. Procedure

**Figure 1:**
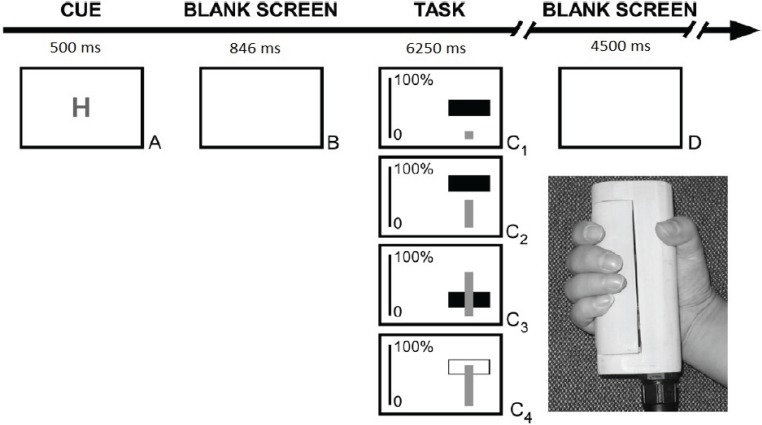
Illustration of the sequence of events and timings in a trial. The picture on the right shows the grip force manipulandum. The grip force is measured by two force sensors embedded in the grip and translated into a signal on screen.

Participants were asked to complete a series of trials in a computer-based motor learning task. The task was a close replication of Kranczioch *et al.* [24]. All stimuli were displayed on black background. Trials began with a cue presented in the middle of the screen for 500 ms indicating the difficulty level (Easy *vs.* Hard) of the forthcoming trial by displaying the letter E or H respectively (Figure 1). After the presentation of a blank screen for 800 ms following the letter E or H, the grip force task began. The task lasted for 5250 ms in each trial. During this part of the trial, a vertical line was shown on screen representing the required target force on a scale from 0% to 100%. In Hard trials the height of the scale was set to 400 pixels; in Easy trials this was set to 200 pixels. Participants were instructed to keep the target force line inside a square by gently adjusting the power grip on a custom-made force manipulandum (see Figure 1) with the right hand. The target square (30 pixels height and 80 pixels width, irrespective of trial difficulty level) represented the target pressure level (target force range, TFR) and was displayed in the central location of the screen. The pressure applied on the grip force measure was represented by a blue vertical bar 10 pixels wide, positioned at the bottom of the screen, in line with the middle point of the target square. By changing the applied force (AF) on the manipulandum, the height of the bar increased or decreased. When the AF was within the target range, *i.e.*, the tip of the blue vertical line was within the target square, the target square turned from red to green and stayed green as long as AF remained within the target range thereby serving as a feedback signal. Participants were instructed to keep the target bar green by adjusting their grip force accordingly.

The target square remained stationary for the first 1000 ms to provide time for participants to adjust the AF to the target force level. For the remaining 4250 ms it followed a set sequence of upward and downward movements. In Hard trials these movements were greater (*ca.* twice the range in pixels), faster (*ca.* double), and more frequent than in easy trials. Application of the same AF resulted in twice as much movement of the line representing the AF on screen in Hard trials compared to Easy trials.

The intertrial-interval (ITI) varied between 4000 ms and 5000 ms with a mean ITI of 4500 ms per block. After each block the percentage of time participants stayed within the target force range for Easy and Hard trials was displayed on the screen to provide performance feedback. Between blocks participants could rest for as long as they preferred.

### 2.3. Transcranial Direct Current Stimulation

1mA tDCS stimulation was applied for twenty minutes using a Magstim^®^ DC stimulator plus (Magstim, Whitland, UK). The current was delivered through two sponged electrode pads (5 cm × 7 cm) soaked in saline solution. The anodal electrode was placed over the left primary motor cortex (M1), identified as electrode position C3 according to the international 10–20 system, and the cathodal electrode over the right supraorbital area. Actual stimulation was preceded by a 20 s ramp-up phase during which the direct current gradually increased to the required level and a similar 20 s ramp-down phase at the end of the stimulation period. In the sham condition the ramp-up phase was immediately followed by the ramp-down phase and the stimulator was then switched off for the remaining 19 min and 20 s of the stimulation phase. In the active tDCS condition the ramp-up phase was followed by 1 mA stimulation for 19 min and 20 s.

### 2.4. Experimental Protocol

**Figure 2:**
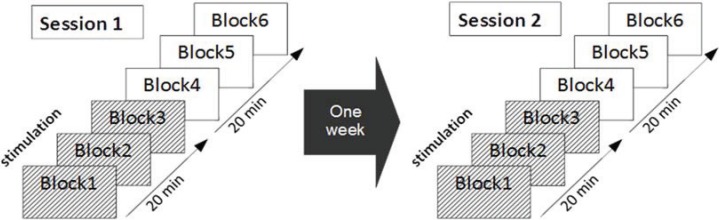
Schematic illustration of the protocol. Each participant received six blocks of task practice. The first three blocks were conducted with tDCS or sham stimulation. Blocks 4–6 were completed during the tDCS afterphase (*i.e.*, the tDCS switched off). Each participant completed two sessions, one with tDCS stimulation and one with sham stimulation (counterbalanced). The sessions were one week apart and controlled for time of day.

The protocol is illustrated in Figure 2. The experiment was run in two sessions one week apart. Each session comprised a practice block and six test blocks. In each test block 15 Easy trials were randomly interspersed with 15 Hard trials. The first three test blocks (Blocks 1–3) were performed under tDCS or Sham stimulation, whereas Block 4 to Block 6 were conducted during the afterphase, *i.e.*, after stimulation (or sham) had ended. The order of active tDCS and sham stimulation across the two sessions was counterbalanced across participants. Thus, eleven participants received tDCS stimulation during the first session and Sham stimulation during the second session, whereas for the other eleven participants this order was reversed. Participants were naive to the stimulation they received.

### 2.5. Data Analysis

Two outcome measures were calculated to assess motor performance: Time-on-target (TOT) and deviation from target (DEV). To create difficulty-specific TOT scores for each participant in each session and block, the number of time-points when the AF was within the target force range, was divided by the total number of time-points in each trial. Next, this ratio was averaged across trials in each block, separately for Easy and Hard trials. DEV scores were calculated in the same fashion, except that the deviation (in pixels) from the target force range at each time-point was divided by the total number of time-points at the beginning, and then were averaged across Hard and Easy trials in each block. These difficulty-specific TOT and DEV scores were used for subsequent statistical analyses.

All statistical analyses were performed with SPSS v20. Repeated measures ANOVAs comprising the factors STIMULATION (tDCS, sham) and BLOCK (1–6), and difficulty (hard, easy) were calculated for TOT and DEV respectively. DEV scores were log-transformed prior to statistical analysis to reduce the skewness of these variables. For the ease of interpretation, untransformed DEV scores are reported in descriptive statistics. Greenhouse-Geisser correction was applied when Mauchly’s test indicated violation of the assumption of sphericity in ANOVA tests. Inspection of the data revealed one outlier with a performance pattern indicating temporary disengagement from the task (*i.e.*, extremely large drop of performance from TOT_hard_ = 0.55 and TOT_easy_ = 0.75 in Block 1 of Session 2 to TOT_hard_ = 0.32 and TOT_easy_ = 0.58 in Block 3 of Session 2. The participant’s performance largely returned to the normal range from Block 4 onwards.). The data of this participant were excluded from further analyses leaving 21 participants for the statistical analyses reported below.

## 3. Results

The analysis addressed the following specific questions. (1) Is there a general learning effect over the course of the experiment? (2) Does anodal tDCS, applied over the contralateral motor cortex, modulate visuomotor performance and learning? (3) Does the performance modulation by tDCS occur during early-learning, defined as performance improvement during the first session, and/or during retention, defined as performance change between block six in Session 1 and block one in Session 2? In addition, we assessed whether tDCS might have differential effects on hard and easy trials. Overall the analysis showed that, while performance clearly improved across the two sessions, tDCS did not affect the learning associated with these performance changes in either phase of the experiment. At the same time, participants reported the tingling sensation typical for tDCS stimulation confirming the successful delivery of the tDCS stimulation. Below the results of the various analyses are reported in detail.

### 3.1. General Learning Effect

**Figure 3:**
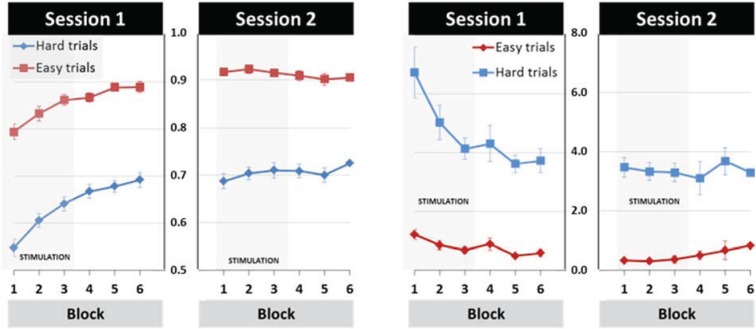
Mean performance for time on target (**left panel**) and the deviance scores (**right panel**) depicted for Hard and Easy trials. The means and standard errors are shown separately for each block and session. The first three blocks (underlayed in grey) were completed with stimulation or sham, blocks 4–6 were completed in the afterphase, *i.e.*, with neither sham nor active tDCS stimulation. The y-axis depicts arbitrary units.

The mean performance data showed considerable performance improvement, which was reflected in increasing TOT and decreasing DEV scores over time. This learning effect was present for hard and easy trials (Figure 3).

Statistically general learning effects were evident by the results from SESSION (first, second) by BLOCK (Blocks 1–6) repeated-measures ANOVAs conducted for Hard and Easy trials and for TOT and DEV respectively. For Hard trials, the main effects of TOT scores were found for SESSION (*F*_(1,20)_ = 45.63, *p* < 0.001, η_p_^2^ = 0.70) and BLOCK (*F*_(5,100)_ = 32.94, *p* < 0.001, *η*_p_^2^ = 0.62) as well as the SESSION × BLOCK interaction (*F*(_5_,_100_) = 24.30, *p* < 0.001, *η*_p_^2^ = 0.55), reflecting greater learning across blocks in the first than in the second session. The same effect pattern was found for the log-transformed DEV scores (SESSION: *F*_(1,20)_ = 16.47, *p* < 0.01, *η*_p_^2^ = 0.45; BLOCK: *F*_(3.27,65.48)_ = 10.33, *p* < 0.001, *η*_p_^2^ = 0.34; SESSION × BLOCK: *F*_(3.20,63.97)_ = 9.10, *p* < 0.001, *η*_p_^2^ = 0.31).

Similar to the Hard trials, the repeated ANOVA of TOT for Easy trials showed significant main effects for SESSION (*F*_(1,20)_ = 39.01, *p* < 0.001, *η*_p_^2^ = 0.66) and BLOCK (*F*_(2.50,49.95)_ = 6.94, *p* < 0.01, *η*_p_^2^ = 0.26) as well as a SESSION × BLOCK interaction (*F*_(5,100)_ = 24.86, *p* < 0.001, *η*_p_^2^ = 0.55). However, in contrast to Hard trials, DEV scores from Easy trials showed a main effect of SESSION (*F*_(1,20)_ = 13.03, *p* < 0.01, *η*_p_^2^ = 0.39) and a SESSION × BLOCK interaction (*F*_(3.27,65.35)_ = 9.41, *p* < 0.001, *η*_p_^2^ = 0.32), but the BLOCK effect was non-significant (*F*_(2.48,49.60)_ = 1.01, *p* = ns, *η*_p_^2^ = 0.05). The absence of a BLOCK effect in the DEV scores for Easy trials is likely due to the fact that Easy trials required smaller and slower changes in force output. Compensatory adjustments are consequently also more controlled causing lower DEV scores. The results of the DEV scores from the Easy trials therefore suggest a ceiling effect on this measure.

To examine the learning effect further, one-way repeated measures ANOVAs of the TOT scores with six levels of BLOCK were conducted separately for each session and difficulty level. The results confirmed that considerable visuomotor learning took place across blocks in the first session, indicated by main effects of BLOCK at both difficulty levels (*F*HARD_(5,100)_ = 57.19, *p* < 0.001, *η*_p_^2^ = 0.74; *F*EASY_(3.12,62.378)_ = 19.971, *p* < 0.001, *η*_p_^2^ = 0.50) for TOT and DEV (*F*HARD_(3.10,61.95)_ = 16.92, *p* < 0.001, *η*_p_^2^ = 0.46; *F*EASY_(2.82,56.31)_ = 5.72, *p* < 0.001, *η*_p_^2^ = 0.22). For the second session, TOT scores showed a small but significant BLOCK effect (*F*_(5,100)_ = 2.93, *p* < 0.05, *η*_p_^2^ = 0.13) for Hard trials but not for Easy trials. DEV scores of the second session were non-significant at both difficulty levels.

Together, these results indicate that visuomotor performance improved considerably over time in both Easy and Hard trials. This performance improvement was largely confined to the first session. In the second session no changes in performance were observed for Easy trials, while performance changes in Hard trials were evident for TOT but not DEV scores. Thereby the TOT effect was small and encompassed an increase of less than 4%. Assuming that these performance changes reflect learning, it follows that tDCS modulation should be strongest in Session 1.

### 3.2. Modulation of Learning and Performance by tDCS Stimulation

**Figure 4:**
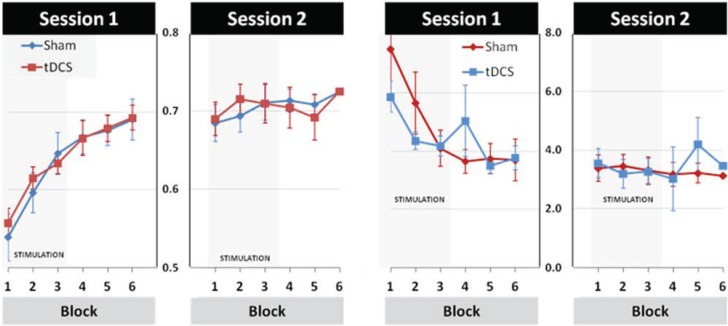
Mean performance in hard trials is depicted per block and session for time-on-target (TOT, **left**) and deviation from target (DEV, **right**). The rate of learning during sham stimulation is similar to the rate of learning during tDCS stimulation suggesting that tDCS has no effect in this particular task. Please note that colours indicate conditions differently for the time-on-target (left) and deviation from target (right) scores.

The effects of tDCS are illustrated in Figure 4. A repeated-measures ANOVA for TOT scores with STIMULATION (tDCS, Sham) and BLOCK (Blocks 1–6) as within-subject factors showed main effects of BLOCK in Hard and Easy trials (*F*HARD_(5,100)_ = 32.94, *p* < 0.001, *η*_p_^2^ = 0.62; *F*EASY_(2.50,49.95)_ = 6.94, *p* < 0.001, *η*_p_^2^ = 0.26). Importantly, however, neither the main effect of STIMULATION (*η*_p_^2^_Hard_ = 0.01, *η*_p_^2^_Easy_ = 0.01), nor the BLOCK × STIMULATION interaction effect (*η*_p_^2^_Hard_ = 0.01, *η*_p_^2^_Easy_ = 0.02) were significant at either difficulty-level. Analysis of the DEV scores revealed a main effect of BLOCK regarding Hard trials *(F*_(3.27,65.48)_ = 10.33, *p* < 0.001, *η*_p_^2^ = 0.34), but not Easy trials (*F*_(2.48,49.60)_ = 1.09, *p* = ns, *η*_p_^2^ = 0.05). Again, there were no significant STIMULATION (*η*_p_^2^_Hard_ = 0.00, *η*_p_^2^_Easy_ = 0.02) or BLOCK × STIMULATION effects for either Hard or Easy trials (*η*_p_^2^_Hard_ = 0.07, *η*_p_^2^_Easy_ = 0.03).

In sum, the results summarised above suggest that, while performance clearly improved with task practice, these effects were not modulated by the tDCS stimulation. Given that some studies specifically reported tDCS effects on early learning while others reported specific tDCS effects on retention, further analyses were conducted to explore these issues.

### 3.3. Effects of tDCS Stimulation on Early Learning and Retention

The results summarised in the section *general learning* indicated that most of the performance improvement took place during the first session only. We therefore conducted an analysis for Session 1 only, where the performance over the six blocks was compared between those participants receiving sham in this session and those receiving active tDCS in this session. No significant differences were found in the performance of these subgroups for either TOT or DEV.

To examine the effects of tDCS on retention, the subgroup data for the last block of session one was compared to the first block of Session 2. Again, no significant effects were found for either TOT or DEV.

## 4. Discussion

In sum, the data of the present study show that, despite the presence of learning effects, the application of 20 min of 1 Hz anodal tDCS, applied to the contralateral motor cortex, did not facilitate this learning and showed no detectable influence on either early learning or retention. The learning effect found in the present study replicates the results obtained in a previous EEG study [24]. The tDCS effects, however, stand in contrast to those studies showing facilitation effects on motor learning but are in line with others reporting null effects of tDCS. These seemingly contradictive findings on tDCS facilitation of motor learning might be explained by differences in the responsiveness of the brain to the neural mechanisms underlying tDCS. Thus, we propose that these mechanisms, and in particular tDCS-facilitated LTP/LTD formation, primarily takes place if the neural system is challenged enough to mandate the functional reorganization of neural networks. For example, this is the case for motor learning in patients with movement disorders. However, for the task employed in the present study, the performance improvements are likely to be mediated through the optimization of neural firing in the motor system as well as changes in the effective connectivity between visual and motor areas. It might therefore be the case that the learning effects observed in the present study are not influenced by tDCS.

An alternative explanation for the tDCS null effect observed here might be to do with the role of area M1 in motor learning more generally. In the present study, the stimulation electrode was placed over M1 using EEG landmarks rather than TMS-based identification. One might therefore argue that the method chosen for electrode positioning was not sufficiently optimized to target the hand representation in M1. To examine this issue, we measured the distance between the C4 electrode position and the first dorsal interosseous muscle (FDI) motor hotspot by employing Transcranial Magnetic Stimulation on 11 participants. The results indicated that the mean distance between the two position is 16.36 mm (*SD* = 6.74), suggesting that the motor hotspot from the chosen EEG position in our study is well within the spatial coverage of the tDCS stimulation electrode. It is therefore reasonable to assume that the tDCS stimulation applied in the present study was successfully delivered to the primary motor cortex. Furthermore, the neural effects of tDCS are spatially relatively diffuse [26] and, applied to M1, tDCS induces excitability changes in various cortical and sub-cortical areas [27]. These are reflected in the BOLD response [28] as well as EEG activity [29]. For instance, Lang *et al.* [27] showed in a positron emission tomography (PET) study that 10 min of 1 mA anodal and also cathodal tDCS induced spatially dispersed and very widespread regional cerebral blood flow changes in the brain, even in regions distant from the stimulation site. Yet given that M1 tDCS triggers significant cortical excitability change in the primary motor cortex [7], our inability to demonstrate tDCS effects could therefore be interpreted as evidence for a limited role of the primary motor cortex in motor learning. This interpretation would be in line with a recent meta-analysis of functional neuroimaging experiments [30], suggesting a much less prominent role of M1 in motor learning than previously thought. Indeed, Nishitani *et al.* [31] showed that the role of M1 did not go beyond motor execution in a visuomotor task and Miall *et al.* [32] proposed that the cerebellum, rather than M1, is the key area for learning visuomotor (eye-hand) coordination. At the same time, other studies have implicated area M1 in the consolidation of motor memories [33] and thus in long-term motor learning. Experimental findings in visuomotor learning support this by showing that offline learning (e.g., between sessions) can be facilitated by M1 tDCS. Reis *et al.* [34] showed that in a visuomotor learning task over several sessions tDCS facilitated learning, but this learning effect was driven by offline learning. Hence they argued that tDCS improves learning by aiding consolidation. Furthermore, Sanes and Donoghue [35] (also Sanes [36]) argue that the primary motor cortex is highly involved in motor learning in general. In line with this concept, previous research showed that visuomotor learning [37] and specifically visuomotor tracking [18] are affected by the application of M1 tDCS. The present study, however, contradicts these findings and, as such, adds to the debate on the specific role of M1 in motor learning and the tDCS modulation thereof.

Performance improvement in the visuomotor task employed here was mostly restricted to the first session, irrespective of whether tDCS was applied or not. Therefore, offline effects of tDCS could be evaluated to a very limited extent only as it seems that participants of both groups reached a ceiling effect by the end of the first session. Our results indicate that tDCS did not influence early learning (*i.e.*, performance improvement within the first session) but, because of the ceiling effect, does not allow conclusions about effects it might have on later visuomotor learning. This also means that studies such as Reis *et al.* [34] visuomotor learning study, which indicated tDCS effects on offline learning, are not necessarily in conflict with our results. It may well be that if the visuomotor task in our study had allowed a more prolonged learning process, *i.e.*, over both sessions, tDCS may have had a positive effect on performance. Furthermore, even though our tracking task in the Hard condition never led to ceiling performance in the absolute sense, most participants reached their maximum achievable performance within the first session. If the task allowed more gradual learning and or had been even more difficult, tDCS stimulation might have been more effective.

Moreover it is also important to consider that currently we do not yet know enough about the factors which might determine why certain participants respond to tDCS whereas others do not. In a recent study, Wiethoff, Hamada and Rothwell [38] showed that 10 min 2 mA anodal stimulation of the motor cortex resulted in no or only minor changes in corticospinal-excitability—Reflected in motor-evoked potential (MEP)—In half of their participants. These results indicate that random variations in sample characteristics may have significant impact on the effectiveness of the employed tDCS stimulation. This individual responsiveness to tDCS in combination with our moderate sample size raises the potential issue, if simply lack of power to detect the tDCS effect could explain our null-finding. Whilst it is plausible to assume that the combination of these factors could have attenuated the effect, it is however important to note that the effect sizes indicated virtually no tDCS stimulation effect in our study. Hence it is likely that the lack of tDCS effect is rooted in other factors than simply lack of statistical power. Another source for discrepancies in the literature is that seemingly small differences in task characteristics can be associated with fundamental differences in the cortical regions involved. There is a complex network of cortical and sub-cortical areas recruited for motor and visuomotor learning with intricate interactions between the various areas. The effect of tDCS on visuomotor learning is necessarily a function of the areas stimulated and the areas involved at various stages of learning during a task. This task-specificity of the tDCS effect was demonstrated by Galea *et al.* [21], showing that cerebellar anodal tDCS improved early learning, whereas anodal tDCS to the contralateral M1 facilitated retention. This suggests that different cortical and sub-cortical areas can be involved at different stages of visuomotor learning of the same task. Furthermore, task-specificity of the tDCS effect is also reflected in the fact that the application of tDCS over a certain brain area can influence different stages of learning in different tasks. For instance, Marquez *et al.* [22] showed that contralateral M1 tDCS modulated early-learning of a sequential finger-tapping task, whereas between session (retention) performance was affected by the same stimulation in a pinch-force task. Similar findings were obtained in a visuomotor tracking task requiring hand-arm coordination [18]. The exact task characteristics therefore might have significant impact on what brain regions are recruited at what stage. It follows that tDCS applied over a certain area might have a very different effect on task performance depending on the procedural details of the task. The latter could also partially explain why the application of tDCS over the M1 resulted in significant performance modulation in some other visuomotor tasks, whilst it had virtually no effect in our study. This also implies that the null-findings of our study should be interpreted with respect to the particular task employed here. Future studies need to establish what task characteristics are important for the effectiveness of tDCS stimulation. For instance, it is possible that even though Reis *et al.* and our tasks are both visuomotor learning tasks, small procedural differences between the studies, such as sequential visual isometric pinch task *versus* precision grip tracking task, may have considerable impact on what neuronal regions are recruited at which stage of the task and therefore what tDCS stimulation specifics are needed to induce a significant modulation of learning.

## 5. Conclusions

Our data suggest that 20 min of 1 mA anodal tDCS, applied to the contralateral motor cortex, does not alter the learning of a task requiring sustained feedback-informed adjustment of the power grip. As such, the findings reported here add to the mixed body of evidence for tDCS facilitation in motor learning. These diverse findings might be due to a number of factors, including differences in task characteristics, as well as more fundamental questions on the functional role of motor cortical regions in motor learning. Moreover, we propose that the susceptibility of neural structures to the mechanisms of tDCS modulation might depend on the level of functional reorganisation required to effectively deliver learning and performance improvement in a task. This might explain why, for example, patients with movement disorders can benefit from the application of therapeutic tDCS in tasks where no effects can be demonstrated for non-clinical populations [39].
